# Supplementary material for: Association between oral microbiome and seven types of cancers in East Asian population: a two-sample Mendelian randomization analysis
Source: Front Mol Biosci. 2023 Nov 21;10:1327893. doi: 10.3389/fmolb.2023.1327893 (PMC10702768; doi:10.3389/fmolb.2023.1327893)
Supplement: Supplementary file 1 [file DataSheet1.ZIP › Supplementary Materials/MR plots of saliva/saliva═╝/Lung Cancer/pheno.2647_to_lung cancer_forest.pdf]

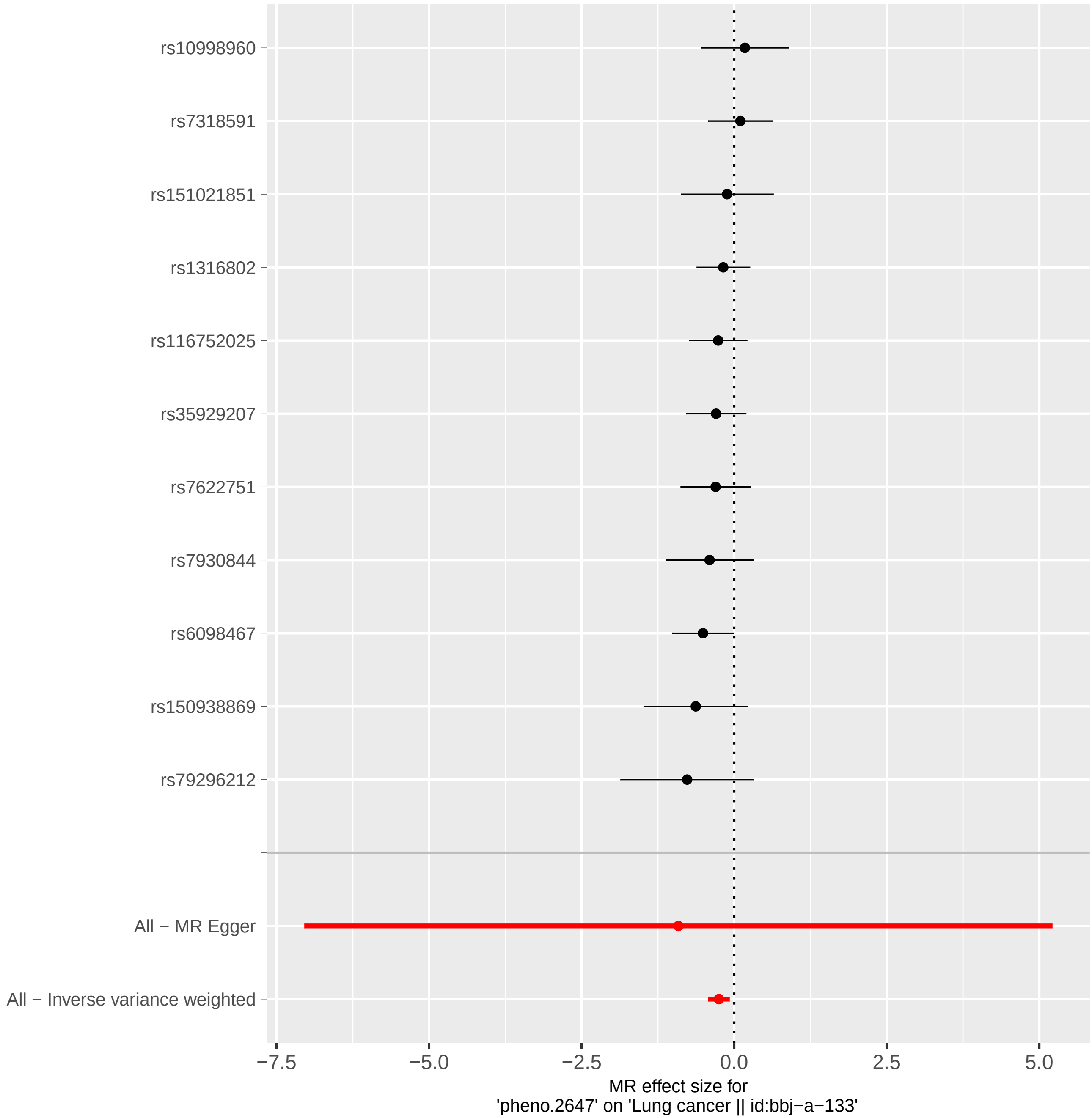

rs10998960
rs7318591
rs151021851
rs1316802
rs116752025
rs35929207
rs7622751
rs7930844
rs6098467
rs150938869
rs79296212
All − MR Egger
All − Inverse variance weighted
−7.5
−5.0
−2.5
0.0
2.5
5.0
MR effect size for
'pheno.2647' on 'Lung cancer || id:bbj−a−133'
